# Hygiene, atopy and wheeze–eczema–rhinitis symptoms in schoolchildren from urban and rural Ecuador

**DOI:** 10.1136/thoraxjnl-2013-203818

**Published:** 2013-10-08

**Authors:** Philip J Cooper, Maritza Vaca, Alejandro Rodriguez, Martha E Chico, Darci N Santos, Laura C Rodrigues, Mauricio L Barreto

**Affiliations:** 1 Laboratorio de Investigaciones FEPIS, Quinindé, Esmeraldas Province, Ecuador; 2 Clinical Sciences, Liverpool School of Tropical Medicine, Liverpool, UK; 3 Escuela de Biología, Pontificia Universidad Católica del Ecuador, Quito, Ecuador; 4 Instituto de Saude Coletiva, Universidade Federal da Bahia, Salvador, Brazil; 5 Department of Epidemiology, London School of Hygiene and Tropical Medicine, London, UK

**Keywords:** Wheeze-Rhinitis-Eczema, Atopy, Hygiene, Farming, Urban-Rural

## Abstract

**Background:**

Rural residence is protective against atopy and wheeze–rhinitis–eczema symptoms in developed countries, an effect attributed to farming and poor hygiene exposures. There are few data from developing countries addressing this question. We compared atopy and wheeze–rhinitis–eczema symptoms between urban and rural Ecuador, and explored the effects of farming and poor hygiene exposures.

**Methods:**

We performed cross sectional studies of schoolchildren living in rural and urban Ecuador. Data on symptoms and farming/hygiene exposures were collected by parental questionnaire, atopy by allergen skin prick test reactivity and geohelminth infections by stool examinations.

**Results:**

Among 2526 urban and 4295 rural schoolchildren, prevalence was: atopy (10.0% vs 12.5%, p=0.06), wheeze (9.4% vs 10.1%, p=0.05), rhinitis (8.1% vs 6.4%, p=0.02) and eczema (5.9% vs 4.7%, p=0.06). A small proportion of symptoms were attributable to atopy (range 3.9–10.7%) with greater attributable fractions for respiratory symptoms observed in urban schoolchildren. Respiratory symptoms were associated with poor hygiene/farming exposures: wheeze with lack of access to potable water; and rhinitis with household pets, no bathroom facilities and contact with large farm animals. Birth order was inversely associated with respiratory symptoms. Area of residence and atopy had few effects on these associations.

**Conclusions:**

Urban schoolchildren living in Ecuador have a similar prevalence of atopy, eczema and wheeze but a higher prevalence of rhinitis compared with rural children. Some farming and poor hygiene exposures were associated with an increase in the prevalence of wheeze or rhinitis while birth order was inversely associated with these symptoms.

**Key messages**

**What is the key question?**

- Do poor hygiene and farming exposures explain the prevalence of atopy and wheeze–rhinitis–eczema symptoms in urban and rural children in a developing country?

**What is the bottom line?**

- The prevalence of rhinitis symptoms but not atopy and wheeze–eczema symptoms was greater in urban compared with rural schoolchildren in tropical Ecuador. Some poor hygiene exposures were associated with an increase in the prevalence of respiratory symptoms while others were associated with less atopy and respiratory symptoms, effects that were largely independent of area of residence.

**Why read on?**

- There are few data from developing countries investigating the effect of poor hygiene and farming exposures on the prevalence of atopy and symptoms of wheeze–rhinitis–eczema. Our data show that environmental exposures indicative of poor hygiene or farming have variable effects on atopy and the risk of respiratory symptoms.

## Introduction

Asthma, rhinitis and eczema are the commonest chronic diseases of childhood in developed countries. A high prevalence of these diseases has been reported in urban centres in Latin America, and the prevalence has been suggested to be lower in rural areas.^1–3^

Several epidemiological studies in developed countries have shown a greater prevalence of asthma in urban compared with rural, essentially farming, populations, and a few studies have shown a similar trend in developing countries.^4^ The protective effects of rural residence have been attributed to farming and hygiene related exposures in developed countries,^4^ ^5^ while in developing countries protective effects have in addition been attributed to the presence of chronic childhood infections, such as geohelminth parasites.^6^ Such protection may be strongest when exposures occur during pregnancy^7^ or early childhood^5^ ^8^ and be mediated through effects on the developing immune response.^5^

There are limited data from developing countries exploring the effects of hygiene and farming exposures on the prevalence of atopy and symptoms of wheeze–rhinitis–eczema in urban and rural populations.^9^ In the present study, we hypothesised that the prevalence of atopy and wheeze–rhinitis–eczema symptoms would be lower in rural compared with urban schoolchildren in Ecuador, and that greater exposure to environmental factors associated with farming and poor hygiene would explain such an effect. We therefore examined the prevalence of atopy and wheeze–rhinitis–eczema symptoms in comparable populations of schoolchildren living in urban and rural areas of the same province in Ecuador, and examined the effects on prevalence of farming and hygiene related factors.

## Methods

### Study area and population

The study was performed in the coastal Province of Esmeraldas, Ecuador, one of the poorest regions of Ecuador with limited public services and infrastructure. The study area was tropical rain forest. The rural area comprised a convenience sample of 59 traditional Afro-Ecuadorian communities along the tributaries of the Santiago river basin in the districts of San Lorenzo and Eloy Alfaro. Economic activities in these communities are logging, subsistence agriculture and African palm oil extraction. The urban study area was the provincial capital of Esmeraldas, the city of Esmeraldas, a city of ∼190 000 inhabitants^10^ whose main economic activities are tourism, services and the oil industry. In the urban area we chose a convenience sample of 11 urban schools in neighbourhoods that contained significant proportions of Afro-Ecuadorian migrants from the same two rural districts who had settled in these neighbourhoods.

### Study design

We did a cross sectional survey of children attending the schools that served the rural communities and the urban neighbourhoods. All children attending the schools at the time of the survey were eligible for inclusion. Based on annually updated school lists, we were able to evaluate 91.3% of children in rural schools and 90.8% of those attending urban schools. Data collection for the rural study was done between March 2005 and August 2008, and for the urban study between September 2008 and January 2010.

### Data collection

#### Questionnaires

The questionnaire was modified from the International Study of Asthma and Allergies in Childhood (ISAAC) phase II questionnaire translated into Spanish, and has been extensively field tested. The questionnaire collected information on symptoms of wheeze, rhinitis and eczema, and risk factors, as described elsewhere,^11^ and is provided as an online archive. The questionnaire was administered to a parent in the presence of the child.

#### Allergen skin prick test reactivity

Allergic sensitisation was measured by skin prick testing with *Dermatophagoides pteronyssinus/farina*e mix, American cockroach (*Periplaneta americana*), *Alternaria tenuis*, cat, dog, ‘9 southern grass mix’ and ‘New stock fungi mix’, and positive histamine and negative saline controls (Greer Laboratories, Lenoir, North Carolina, USA), as described previously.^11^ A positive reaction was defined as a mean wheal diameter of at least 3 mm greater than the saline control at 15 min. The same observer performed all skin prick testing (MV).

#### Stool examinations

Single stool samples were collected and analysed for geohelminth eggs and larvae using the modified Kato Katz and formol**–**ether concentration methods.^12^

### Definition of outcomes

Outcomes were defined as: atopy—the presence of at least one positive allergen skin test; recent wheeze—reported wheezing during the previous 12 months; recent eczema—having a reported itchy rash with a flexural distribution in the previous 12 months; and recent rhinitis—nasal stuffiness or sneezing without a cold accompanied by itchy eyes in the previous 12 months.

### Statistical analysis

Sample sizes of 2500 for the urban and 4000 for the rural studies were estimated to yield approximately 200 asthma cases for nested case control studies in each area. Associations between poor hygiene/farming exposures and study outcomes were explored using univariate and multivariate random effects logistic regression adjusted for clustering by community or neighbourhood. Exposures in multivariate models were selected using a backwards stepwise procedure in which exposures were included if p<0.2 or OR ≥10%. Interactions by area of residence or atopy were assessed using the Wald test. Because of multiple comparisons, we used a p value ≤0.01 as evidence for effect modification or of a variable being statistically significant in multivariate models. Population attributable fractions (PAF) were calculated by: P_ew_×(OR**−**1)/OR, where P_ew_ is the prevalence of allergen skin test reactivity among children with the specific symptom of interest. Analyses were done using STATA (V.10).

### 

Written informed consent was obtained from a parent, and signed minor assent from the child. Appropriate antiparasitic treatment was offered where necessary.

## Results

### Characteristics of urban and rural schoolchildren

**Figure 1:**
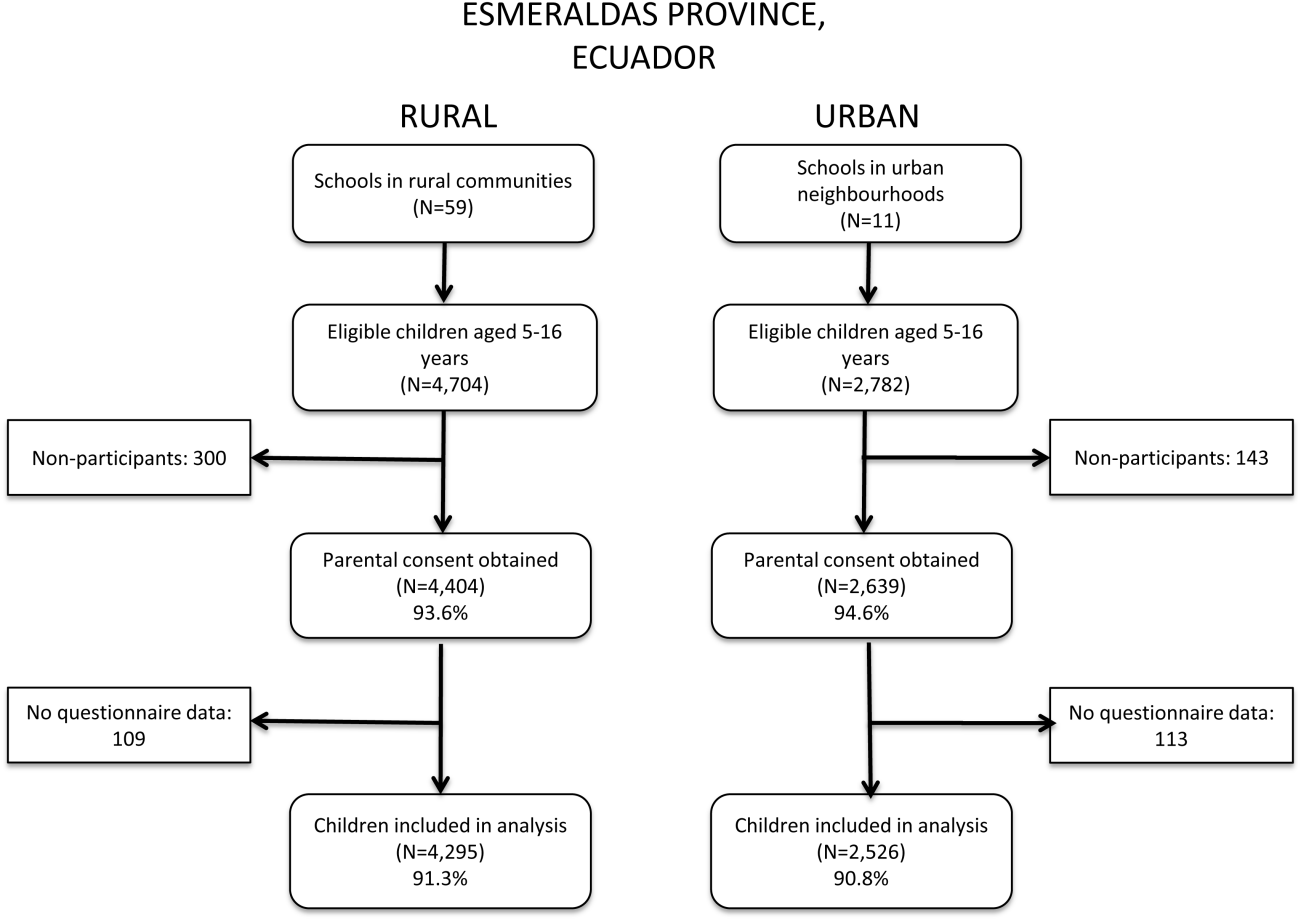
Flow diagram showing recruitment of schoolchildren in urban and rural areas.

We studied a total of 6821 schoolchildren in urban (2526) and rural (4295) areas. Recruitment of the study subjects is shown in figure 1 and the distributions of risk factors between urban and rural schoolchildren in table 1. Rural compared with urban children were slightly older (p<0.001), more likely to be Afro-Ecuadorian (p<0.001), have less educated mothers (p<0.001) and a lower household income (p<0.001), be underweight (p<0.001) and higher in the birth order (p<0.001), not have access to a bathroom for defecation (p<0.001) or access to potable drinking water (p<0.001), to have attended daycare (p=0.008), to have a father engaged in agriculture (p<0.001) and have contact with large farm animals (p<0.001), to consume unpasteurised milk (p<0.001) and have a higher prevalence of *Ascaris lumbricoides* (p<0.001) and *Trichuris trichiura* (p<0.001).

**Table 1:** Characteristics of schoolchildren in urban and rural areas

Risk factor	Urban (n=2526)		Rural (n=4295)		p Value for rural vs urban
	n	%	n	%	
*Demographics*					
Age (years)					
5–8	856	33.9	1197	27.9	<0.001
9–11	1252	49.6	1511	35.2	
12–16	418	16.5	1587	36.9	
Sex					
Female	1196	47.4	2089	48.6	0.303
Male	1330	52.6	2206	51.4	
Ethnicity*					
Afro-Ecuadorian	2116	83.9	3957	92.5	<0.001
Other	405	16.1	323	7.5	
Maternal educational level					
Illiterate	546	22.0	2434	56.8	<0.001
Completed primary	1257	50.0	1498	35.0	
Completed secondary	720	28.0	353	8.2	
Monthly income (US$)					
≤150	968	39.2	3370	79.9	<0.001
>150	1499	60.8	845	20.1	
Nutritional status					
Underweight	341	13.5	703	16.4	<0.001
Normal	1718	68.0	2976	69.3	
Overweight	467	18.5	616	14.3	
*General hygiene factors*					
Pets inside the house					
No	1004	39.7	1650	38.5	0.293
Yes	1522	60.3	2640	61.5	
Crowding (persons/sleeping room)					
<Median	1298	51.5	2141	49.9	0.202
>Median	1222	48.5	2149	50.1	
Birth order					
≥5th	1367	54.2	1726	40.2	<0.001
3rd–4th	727	28.8	1119	26.1	
1st–2nd	426	17.0	1450	33.7	
Bathroom (%)					
Field	178	7.1	1553	36.1	<0.001
Latrine	595	23.5	2617	61.0	
WC	1752	69.4	123	2.9	
Potable drinking water					
No	199	7.9	4028	93.8	<0.001
Yes	2320	92.1	266	6.2	
Daycare attendance					
No	1431	57.4	2291	54.1	0.008
Yes	1061	42.6	1944	45.9	
Household construction					
Wood/bamboo	218	8.7	2443	57.2	<0.001
Mixed cement/wood	1139	45.2	1151	26.9	
Cement	1163	46.1	678	15.9	
*Farming exposures*					
Father with agricultural occupation					
No	2306	93.8	2213	52.4	<0.001
Yes	152	6.2	2007	47.5	
Contact with large farm animals*					
No	2306	91.4	2979	69.5	<0.001
Yes	218	8.6	1310	30.5	
Unpasteurised milk†					
No	1594	63.2	2455	57.3	<0.001
Yes	927	36.8	1831	42.7	
*Infections*					
Geohelminth infections					
Any geohelminth	1036	42.9	2849	69.0	<0.001
*Ascaris lumbricoides*	479	19.9	1752	42.4	<0.001
*Trichuris trichiura*	853	35.4	2234	54.1	<0.001
*Hookworm*	111	4.6	228	5.5	0.106

Median crowding was 3.

Numbers of missing values (rural/urban) are given in parentheses: ethnicity (15/5); maternal educational level (10/3); monthly income (80/59); pets inside the house (5/0); crowding (5/6); birth order (0/6); bathroom (2/1); potable drinking water (1/7); daycare attendance (60/34); household construction (23/6); father with agricultural occupation (75/68); contact with large farm animals (6/2); unpasteurised milk (9/5); any geohelminth (163/113); *Ascaris lumbricoides* (163/113); *Trichuris trichiura* (163/113); Hookwor*m* (163/113).

*Pigs, cows, horses, mules, donkeys.

†Consumption of unpasteurised milk at least once weekly.

### Prevalence of atopy and symptoms of wheeze–rhinitis–eczema

The prevalence of atopy and recent symptoms of wheeze–rhinitis–eczema symptoms in the urban and rural samples is shown in table 2. The prevalence of wheeze was slightly greater in rural (10.1%) compared with urban (9.4%) schoolchildren (p=0.05). There were no differences in the prevalence of other wheeze related symptoms and markers of wheeze severity between urban and rural schoolchildren (data not shown). The prevalence of rhinitis symptoms (with itchy eyes) was significantly higher in urban schoolchildren (p=0.02). There was some evidence for a higher prevalence of eczema symptoms (itchy flexural rash) in the urban sample (p=0.06). The prevalence of allergen skin prick test reactivity (SPT) tended to be greater in rural children (urban 10.0% vs rural 12.5%, p=0.06), a difference largely explained by a higher prevalence of SPT to American cockroach (p<0.001) and dog (p<0.001) in rural children.

**Table 2:** Frequencies of symptoms and atopy in 6821 schoolchildren living in urban and rural areas of Esmeraldas Province

Variable	Urban (n=2526)		Rural (n=4295)		OR (95% CI)	p Value
	n	%	n	%		
Wheeze						
Wheeze ever	801	32.7	1362	32.7	0.97 (0.87 to 1.09)	0.64
Recent wheeze	231	9.4	421	10.1	0.84 (0.71 to 1.00)	0.05
Rhinitis						
Rhinitis ever	489	19.5	553	13.1	**1.57 (1.37 to 1.80)**	<0.001
Recent rhinitis	203	8.1	270	6.4	**1.25 (1.03 to 1.52)**	0.02
Eczema						
Eczema ever	323	12.9	358	8.4	**1.57 (1.33 to 1.84)**	<0.001
Recent eczema	146	5.9	199	4.7	1.24 (1.00 to 1.56)	0.06
Skin prick test reactivity						
Any allergen	246	10.0	515	12.5	0.85 (0.72 to 1.01)	0.06
House dust mite	185	7.6	281	6.8	1.19 (0.97 to 1.44)	0.09
Mixed grass	27	1.1	76	1.8	0.68 (0.43 to 1.07)	0.10
American cockroach	55	2.2	185	4.5	**0.54 (0.40 to 0.74)**	<0.001
Mixed fungi	10	0.4	18	0.4	1.31 (0.58 to 2.94)	0.52
*Alternaria tenuis*	3	0.1	8	0.2	0.67 (0.17 to 2.60)	0.456
Cat	8	0.3	17	0.4	0.84 (0.36 to 1.98)	0.69
Dog	6	0.2	68	1.7	**0.15 (0.07 to 0.35)**	<0.001

ORs and 95% CIs show urban and rural comparisons adjusted for age and sex.

Numbers in bold represent p<0.05.

Recent represents symptoms within the previous 12 months.

Eczema was defined by an itchy rash with a flexural distribution and rhinitis by nasal stuffiness/sneezing with itchy eyes.

### Associations between atopy and symptoms of wheeze–rhinitis–eczema

The associations between recent symptoms and SPT in urban and rural schoolchildren are shown in table 3. Wheeze (p<0.001) and rhinitis (p=0.004) were significantly associated with SPT in urban children while among rural children a significant association was seen for wheeze only (p=0.04). The associations between atopy and wheeze or rhinitis were significantly stronger in urban than rural children (interaction p=0.01). Recent eczema (itchy flexural rash) was weakly associated with SPT (urban, p=0.06; rural, p=0.05). A small fraction of symptoms were attributable to SPT (table 3): population attributable fractions for wheeze in urban and rural children were 10.7% and 3.9%, respectively, and for rhinitis and eczema were <10% in both areas.

**Table 3:** Associations between recent symptoms and allergen skin prick test reactivity and population fractions of symptoms attributable to skin prick test reactivity (PAF%) in urban and rural schoolchildren

Recent symptoms	SPT						Interaction p value
	Urban			Rural			
	OR (95% CI)	p Value	PAF%	OR (95% CI)	p Value	PAF%	
Wheeze	**2.35 (1.63 to 3.40)**	**<0.001**	10.7	**1.36 (1.01 to 1.83)**	**0.04**	3.9	0.01
Rhinitis	**1.82 (1.22 to 2.73)**	**0.004**	7.3	0.94 (0.63 to 1.40)	0.75	–	0.01
Eczema	1.60 (0.99 to 2.60)	0.06	5.7	1.47 (1.00 to 2.16)	0.05	5.3	0.74

Shown also are p values for the interaction effect of the area of residence.

Numbers in bold represent p<0.05.

ORs are adjusted for age, sex and maternal educational level (eczema only).

PAF%, population attributable fraction; SPT, skin prick test reactivity.

### Associations between hygiene/farming exposures and atopy and wheeze–rhinitis–eczema symptoms

We explored the effects of poor hygiene and farming exposures on study outcomes (table 4; frequencies are provided in the online supplementary table S1). Multivariate analyses controlled simultaneously for the effects of these exposures: SPT was inversely associated with *A lumbricoides* (p=0.002) and *T trichiura* (p<0.001) infections; wheeze was positively associated with lack of potable drinking water (p=0.001) and inversely associated with being higher in the birth order (p=0.005); rhinitis was positively associated with household pets (p=0.004), lack of household bathroom facilities (p=0.003) and contact with farm animals (p=0.001) but inversely with birth order (p<0.001); eczema was not associated with any of the exposures.

**Table 4:** Univariate and multivariate associations between outcomes and hygiene exposures in 6821 schoolchildren

Hygiene exposure	SPT OR (95% CI) p Value		Wheeze OR (95% CI) p Value		Rhinitis OR (95% CI) p Value		Eczema OR (95% CI) p Value	
	Univariate	Multivariate	Univariate	Multivariate	Univariate	Multivariate	Univariate	Multivariate
Pets inside home Yes vs no	0.91 (0.78 to 1.06) 0.215		1.09 (0.93 to 1.29) 0.296		1.34 (1.09 to 1.62) 0.005	**1.34 (1.09 to 1.63)** **0.004**	1.28 (1.03 to **1**.60) 0.031	1.19 (0.94 to 1.51) 0.151
Crowding ≥3 vs <3	0.79 (0.67 to 0.92) 0.004		0.97 (0.82 to 1.16) 0.733		0.86 (0.70 to 1.05) 0.133	0.81 (0.66 to 1.0) 0.053	0.88 (0.71 to 1.11) 0.276	
Birth order ≥5th vs ≤4th	1.58 (0.98 to 1.37) 0.084		0.75 (0.62 to 0.91) 0.003	**0.75 (0.61 to 0.91)** **0.005**	0.62 (0.49 to 0.78) <0.001	**0.62 (0.49 to 0.79)** **<0.001**	0.79 (0.62 to 1.02) 0.069	0.78 (0.60 to 1.02) 0.070
Bathroom Field vs others	0.91 (0.76 to 1.09) 0.299		1.19 (0.99 to 1.43) 0.054		1.32 (1.08 to 1.62) 0.008	**1.44 (1.13 to 1.83)** **0.003**	0.80 (0.62 to 1.04) 0.097	
Potable drinking water No vs yes	1.05 (0.81 to 1.34) 0.700		1.21 (1.02 to 1.43) 0.028	**1.44 (1.16 to 1.78)** **0.001**	0.92 (0.72 to 1.20) 0.562	0.79 (0.59 to 1.06) 0.123	0.96 (0.78 to 1.20) 0.771	
Attended daycare Yes vs no	0.96 (0.82 to 1.12) 0.642		0.99 (0.84 to 1.16) 0.889		1.11 (0.92 to 1.33) 0.296		1.28 (1.4 to 1.59) 0.023	1.28 (1.02 to 1.60) 0.037
House construction Wood/bamboo vs others	1.14 (0.98 to 1.33) 0.087		0.95 (0.81 to 1.12) 0.508		1.12 (0.93 to 1.35) 0.242	1.28 (1.03 to 1.61) 0.027	1.02 (0.83 to 1.26) 0.854	
Father engaged in agriculture Yes vs no	1.55 (1.32 to 1.81) <0.001	1.24 (1.01 to 1.51) 0.036	0.89 (0.74 to 1.06) 0.176	0.86 (0.70 to 1.05) 0.144	0.82 (0.66 to 1.01) 0.062	0.89 (0.69 to 1.15) 0.386	1.24 (0.99 to 1.56) 0.055	
Contact with farm animals* Yes vs no	1.40 (1.18 to 1.66) <0.001	1.15 (0.95 to 1.41) 0.146	1.03 (0.85 to 1.25) 0.781		1.28 (1.04 to 1.59) 0.021	**1.50 (1.19 to 1.91)** **0.001**	1.38 (1.09 to 1.75) 0.008	1.41 (1.07 to 1.84) 0.014
Unpasteurised milk† Yes vs no	1.13 (0.97 to 1.32) 0.109		1.05 (0.89 to 1.24) 0.557		1.02 (0.85 to 1.24) 0.810		1.34 (1.08 to 1.65) 0.008	1.13 (0.89 to 1.43) 0.321
Any geohelminth Yes vs no	0.66 (0.56 to 0.77) <0.001		1.12 (0.95 to 1.34) 0.165		0.94 (0.78 to 1.14) 0.550		0.97 (0.78 to 1.21) 0.810	1.31 (0.93 to 1.85) 0.125
*Ascaris lumbricoides* Yes vs no	0.71 (0.59 to 0.84) <0.001	**0.73 (0.60 to 0.89)** **0.002**	1.01 (0.84 to 1.20) 0.924		0.83 (0.68 to 1.02) 0.079		1.03 (0.82 to 1.29) 0.810	
*Trichuris trichiura* Yes vs no	0.63 (0.53 to 0.73) <0.001	**0.71 (0.59 to 0.85)** **<0.001**	1.16 (0.98 to 1.36) 0.084		1.07 (0.88 to 1.29) 0.505		0.78 (0.63 to 0.97) 0.025	0.72 (0.51 to 1.02) 0.061
Hookworm Yes vs no	1.38 (1.01 to 1.88) 0.047	1.37 (0.95 to 1.97) 0.088	1.10 (0.77 to 1.58) 0.591		0.74 (0.46 to 1.20) 0.221		1.37 (0.90 to 2.12) 0.143	

Variables in multivariate analyses with p≤0.01 were considered statistically significant and are shown in bold type.

Median crowding was 3.

*Pigs, cows, horses, mules, donkeys.

†Consumption of unpasteurised milk at least once weekly.

SPT, skin prick test reactivity.

### Effect modification by area or residence and atopy on associations between poor hygiene/farming exposures and wheeze–rhinitis–eczema symptoms

We explored the effects of area of residence or atopy on the associations between study exposures and wheeze–rhinitis–eczema symptoms. Complete results are provided in online supplementary tables S2 and S3. Area of residence modified the association between birth order and wheeze, with an inverse association seen only in rural schoolchildren (urban, adjusted OR 1.13 (95% CI 0.79 to 1.81, p=0.497) vs rural, adjusted OR 0.63 (95% CI 0.50 to 0.80, p<0.001), interaction p=0.007). For atopy, there was evidence of a significantly greater prevalence of wheeze among non-atopic children without access to potable drinking water (SPT−, adjusted OR 1.42, 95% CI 1.13 to 1.79, p=0.002; SPT+, adjusted OR 0.74, 95% CI 0.47 to 1.14, p=0.172; interaction p=0.01). Non-atopic children living in traditionally built houses had a greater prevalence of rhinitis compared with atopics (SPT−, adjusted OR 1.37, 95% CI 1.09 to 1.72, p=0.008; SPT+, adjusted OR 0.66, 95% CI 0.36 to 1.21, p=0.179; interaction p=0.005) while atopic children without access to potable drinking water had a reduced risk of rhinitis compared with non-atopics (SPT+, adjusted OR 0.48, 95% CI 0.26 to 0.88, p=0.019; SPT−, adjusted OR 1.05, 95% CI 0.79 to 1.40, p=0.741; interaction p=0.003). No effect modification by atopy was observed for eczema.

## Discussion

In the present study of schoolchildren living in urban and rural areas of a tropical region in Latin America, we did not observe a significantly greater prevalence of atopy, wheeze and eczema symptoms in urban compared with rural samples although there was a greater prevalence of rhinitis symptoms in urban schoolchildren. The strength of the association between atopy and respiratory symptoms was greater in urban children. Some poor hygiene/farming exposures were associated with an increased risk of respiratory symptoms, while being higher in the birth order was inversely associated. There was limited evidence for modification of these effects by urban versus rural residence or atopy. Our data emphasise the fact that rural children do not necessarily have a reduced prevalence of atopy and wheeze–rhinitis–eczema symptoms compared with urban children, and that some exposures indicative of poorer hygiene may increase the risk of respiratory symptoms.

A previous study comparing population samples of individuals living in rural subsistence communities with those in a non-industrial urban environment showed a higher prevalence of atopy to house dust mite in the rural but more wheeze symptoms in the urban population.^13^ Since then, other studies have reported an elevated prevalence of asthma and atopy in urban compared with rural populations in both developing and developed countries.^4^ ^14^ An urban–rural effect on risk of eczema is less consistent although a systematic review suggested that the prevalence of eczema may be increased in some urban populations.^15^

So why did we not observe such differences in the present study? We have shown previously that there is significant heterogeneity in the level of urbanisation between rural communities in the rural area where we conducted the study and that a higher level of urbanisation, particularly the adoption of a more urban lifestyle, was associated with the prevalence of wheeze at the community level.^16^ Thus there is considerable heterogeneity in asthma risk between rural communities, and considering all as a single entity will mask these differences. Urban residence in the present study, although associated with changes in the living environment and presumably other factors such as exposure to air pollution, was still associated with significant rural exposures, such as farming. Such exposures likely reflect the lifestyle of more recent rural migrants who maintain rural lifestyles and contacts with their origins. Our urban study population can, therefore, be considered at a relatively early stage in the transition to an urban way of life, and such changes as do occur did not translate into significant changes in the prevalence of atopy, wheeze and eczema. Perhaps the first changes to occur in allergy during this early stage of urban transition are an increase in the prevalence of rhinitis and a strengthening of the association between atopy and respiratory symptoms, as observed here.

The rural area where we conducted this study represents traditional rural communities that have just started the transition to a more modern way of living. Many were accessible only by river, were not connected to the national electricity grid and used traditional materials for housing. But no community was truly isolated from urban influences—many rural residents had travelled to urban centres and the economies of all communities were money based. Most agriculture in these rural communities was subsistence. The urban study population was chosen to be representative ethnically and socially of the rural population, and urban study neighbourhoods were located at the periphery of the city of Esmeraldas where some but limited basic services were present. Such marginal populations living at the periphery of small to medium sized cities is expected to fuel much of the growth of the world population in the 21st century.

The hygiene hypothesis developed from the observation of an inverse association between sibling number and rhinitis that was explained by unhygienic contacts with older siblings.^17^ Since then, this hypothesis has been extended to include the effects of a wide variety of infectious and other microbial exposures (eg, farming and pets in affluent countries and parasites in non-affluent countries) on a wide range of inflammatory diseases, extending from allergic to autoimmune diseases.^18^ A plausible underlying mechanism to explain such wide ranging effects is the induction of immune regulation through the production of regulatory cytokines, such as interleukin 10 that serves to modulate Th1 and Th2 mediated inflammation.^18^ Several environmental exposures associated with poor hygiene, including geohelminths, have been associated with increased interleukin 10,^19^ ^20^ providing a biologically plausible link between chronic microbial exposures and reduction in tissue inflammation. Consistent with such a paradigm, we observed a reduced prevalence of SPT among children with geohelminths. Being higher in the birth order was inversely associated with respiratory symptoms, in agreement with previous studies,^17^ ^21^ but other exposures representative of farming, poor hygiene or increased risk of infections (ie, lack of potable drinking water) were associated with an increased risk of these symptoms.

Previous observations from urban Brazil showed strong associations between non-atopic wheeze and indicators of dirt, increased urban poverty and respiratory infections.^22^ Although such observations are not consistent with the hygiene hypothesis, increased exposure to chronic parasitic, bacterial and viral infections were strongly inversely associated with atopy.^23^ In our study, only a minority of wheeze–rhinitis–eczema symptoms were attributable to atopy (<11%), in agreement with previous studies from Latin America.^2^ ^24–26^ A study of European children indicated that the effects of hygiene exposures on non-atopic symptoms were distinct from those on atopic symptoms.^27^ In the present study, there was some evidence to suggest that poor hygiene exposures might increase the prevalence of respiratory symptoms in non-atopic compared with atopic schoolchildren.

Being born and raised on a livestock farm in Europe provides the strongest protection against atopy and allergic symptoms.^5^ Such protection may require an intimate relationship between herd animals and their owners, particularly during the winter months when the animals may be kept in barns close to the farmer's living space and where exposures to the animals and their microbes is intense, perhaps leading to immune tolerance and reduced inflammation. Clearly not all farm exposures are protective, and significant heterogeneity of effects has been reported across Europe,^28^ while an increased risk has been reported in Australasian^29^ ^30^ and Iowan children.^31^ The relationship between farming families and their animals is different in the tropical lowlands of Ecuador and a less intimate association could explain why childhood contact with large farm animals, through exposures to farming related irritants or proinflammatory substances, might be associated with an increase in rhinitis and perhaps also eczema symptoms.

The strengths of the present study were: evaluation of a large population of urban and rural schoolchildren within the same geographic region in Ecuador with sufficient power to detect relevant effects of exposures on atopy and allergic symptoms; very high rates of participation in both study areas (>90%) ensuring the relevance of our findings to the largely Afro-Ecuadorian population we studied; and use of simple and widely used measurements of parentally reported symptoms. The use of symptoms to estimate the prevalence of wheeze–rhinitis–eczema is probably subject to less bias than a doctor diagnosis in a population with limited access to healthcare and where such access differs between urban and rural populations. The term wheeze–rhinitis–eczema was used as a substitute for ‘allergic’ because few such symptoms in our study population appear to be explained by atopy. Weaknesses were a lack of data on the intensity and age of initial exposures to these factors, and the questionnaire data may have been subject to recall bias and misclassification. We used SPT rather than allergen specific IgE to measure atopy and cannot comment on the effects of the study exposures on this latter marker. However, SPT, a measure of allergic effector responses rather than allergic sensitisation per se, may be more appropriate for the exploration of such effects in a population in which the two atopic markers are dissociated.^23^ ^26^

In summary, the present study, performed in rural and urban schoolchildren in Ecuador, showed a greater risk of rhinitis symptoms in urban compared with rural children but no such effects on the prevalence of wheeze and eczema. There was evidence that some poor hygiene/farming exposures were associated with an increased risk of respiratory symptoms, while being higher in the birth order was protective. Our observations provide further insights into the determinants of the prevalence of atopy and wheeze–rhinitis–eczema symptoms in a population in Latin America undergoing development and at an early stage in the evolution of the so-called allergy epidemic that has emerged over recent years in more advanced Latin American countries and in other developing regions.

## Supplementary Material

Web Questionnaire

Web tables
