# Supplementary material for: Hygiene, atopy and wheeze–eczema–rhinitis symptoms in schoolchildren from urban and rural Ecuador
Source: Thorax. 2013 Oct 8;69(3):232–9. doi: 10.1136/thoraxjnl-2013-203818 (PMC3932750; doi:10.1136/thoraxjnl-2013-203818)
Supplement: Web Questionnaire [file thoraxjnl-2013-203818-s1.pdf]

1. NUMERO…………………

CUESTIONARIO

FACTORES DE RIESGO PARA ALERGIAS Y ASMA

2. Fecha.............../................./200

3.Apellidos/Nombres delJefedela casa..

4. Número de casa por Censo de Oncocercosis............................

5. Apellidos y Nombres del entrevistador..

A. Datos personales

6. Apellidos y Nombres (del niño)……………………………………………………..

7. Edad..........años **8. Fecha de nacimiento**.........../.........../...........

9. Raza: Afro-ecuatoriana ❑ **1** Mestiza ❑ **2** Indígena ❑ **3**

10.Localidad donde vive el niño…………………... **11. Provincia**.......................

12. Escuela... No estudia ❑

13. Paralelo: ....de EB No sabe ❑

14. Sexo: F ❑ **2** M ❑ **1**

B. Datos Socio-Económicos

19. La relación entre el niñ(a) y el JEFE de la casa es: Ausente ❑ **0**

Padre ❑ **1** Padrastro ❑ **2** Abuelo ❑ **3** Tío ❑ **4** Otro **5**..................

20. Ocupación del JEFE de la casa (especificar):

Jornalero	❑ **1**	Comerciante	❑ **6**	Chofer	❑ **11**
Agricultor	❑ **2**	Profesor	❑ **7**	Desempleado	❑ **12**
Ganadero	❑ **3**	Carpintero	❑ **8**	No sabe	❑ **99**
Motorista	❑ **4**	Albañil	❑ **9**		
Maderero	❑ **5**	Mecánico	❑ **10**	Otros **13** (especifique)	_____________

Firma……………………. **Fecha…../……./200**

1. NUMERO…………………

21. Instrucción del JEFE de la casa:

primaria completa	❑ **1**	primaria incompleta	❑ **5**
secundaria completa	❑ **2**	secundaria incompleta	❑ **6**
superior completa	❑ **3**	superior incompleta	❑ **7**
analfabeto	❑ **4**	No sabe	❑ **99**

22. Lugar de nacimiento del jefe de la casa:
....(localidad/ ciudad/ provincia)

23. El jefe de la casa se crió en zona: urbana ❑ **1** rural ❑ **2** No sabe ❑ **99**

24. ¿Actualmente el jefe de la casa vive en una zona (rural o urbana) distinta a la que se crió?

Si ❑ **1** No ❑ **0** No sabe ❑ **99**

☛ **Si la respuesta a la pregunta 24 es <u>SI,</u> indique si el jefe de la casa ha migrado de:**

25. Zona rural a urbana Si ❑ **1** No ❑ **0**

26. Zona urbana a rural Si ❑ **2** No ❑ **0**

☛ **Si el jefe de la casa ha migrado especificar la ocupación que tuvo antes de migrar:**

27. (De zona rural a urbana) ______________________

28. (De zona urbana a rural) ______________________

29. La relación entre el niño(a y la SEÑORA de la casa es: Ausente ❑ **0**

Madre ❑ **1** Madrastra ❑ **2** Abuela ❑ **3** Tía ❑ Otro **5**.................

30. **Ocupación de la SEÑORA de la casa (especificar):**

Quehaceres domésticos	❑ **1**	Profesora	❑ **6**	Desempleada	❑ **11**
Empleada doméstica	❑ **2**	Comerciante	❑ **7**	No sabe	❑ **99**
Jornalera	❑ **3**	Cocinera	❑ **8**		
Agricultora	❑ **4**	Partera	❑ **9**		
Lavandera	❑ **5**	Costurera	❑ **10**		

Otros (especifique) **12** ____

Firma…………………….　　　　　　　　　　**Fecha…../……./200**

1. NUMERO…………………

31. Instrucción de la SEÑORA de la casa:

primaria completa	❐ **1**	primaria incompleta	❐ **5**
secundaria completa	❐ **2**	secundaria incompleta	❐ **6**
superior completa	❐ **3**	superior incompleta	❐ **7**
analfabeta	❐ **4**	No sabe	❐ **99**

32. Lugar de nacimiento de la señora de la casa?

....(localidad/ ciudad/ provincia)

33. La señora de la casa se crió en zona: urbana ❐ **1** rural ❐ **2** No sabe ❐**99**

34. ¿Actualmente si la señora de la casa vive en una zona (rural o urbana) distinta a la que se crió?

Si ❐ **1** No ❐ **0** No sabe ❐ **99**

☛ **Si la respuesta a la pregunta 34 es SI, indique si la señora de la casa ha migrado de:**

35. Zona rural a urbana Si ❐ **1** No ❐ **0**

36. Zona urbana a rural Si ❐ **1** No ❐ **0**

☛ **Si la señora de la casa ha migrado especificar la ocupación que tuvo antes de migrar:**

37. (De zona rural a urbana) ________________________

38. (De zona urbana a rural) ________________________

39. ¿Cuánto es el ingreso mensual familiar? $................
(suma total del dinero que aportan todas las personas que viven en la casa)

40. Vive en casa: propia ❑1 arrendada ❑ 2 prestada ❑3

Firma……………………. **Fecha…../……./200**

1. NUMERO…………………

41. ¿Qué tipo de construcción tienen las paredes de la casa en la que vive el Niño(a)?

Madera	❑1	Ladrillo/ bloque/ cemento	❑4
Mixta (madera/ caña)	❑2	Mixta (madera /cemento)	❑5
Caña	❑3	Otro (especifique)	❑ 6 ____________

42. ¿Cuántos cuartos (no se incluye los baños) existen en la casa? ....

43. ¿Cuántos dormitorios existen en la casa? ....

44. ¿Cuántas personas viven/ duermen permanentemente en la casa? ....

45. Usted utiliza:

Letrina	❑1	Servicio higiénico	❑2	Campo	❑3	Otro (especifique)	4 ____________

46. ¿El baño lo comparte con otras familias? : Si ❑ **1** No ❑ **0**

47. **¿Usted tiene Luz?** Si ❑ **1** No ❑ **0**

☛ **¿Qué artefactos eléctricos hay en la casa?**

**48.** Refrigeradora	Si ❑ **1**	No ❑ **0**	**49.** Televisión	Si ❑ **1**	No ❑ **0**
**50.** Equipo de sonido	Si ❑ **1**	No ❑ **0**	**51.** Radio	Si ❑ **1**	No ❑ **0**

☛ **¿Qué material utiliza para cocinar?**

**52.** Gas	Si ❐ **1** No ❐ **0**	**53.** Carbón	Si ❐ **1** No ❐ **0**
**54.** Leña	Si ❐ **1** No ❐ **0**	**55.** Otro (especifique)	________________

☛ **¿En su casa tiene?**

56. Carro **Si** ❐ **1** **No** ❐ **0**

57. Canoa a motor **Si** ❐ **1** **No** ❐ **0**

Firma……………………. **Fecha…../……./200**

1. NUMERO…………………

☛ **¿Cuáles son las fuentes principales de recolección de agua?**

**58.** Potable	**Si** ❐**1** **No**❐**0**	**59.** Entubada	**Si** ❐ **1** **No**❐ **0**	**60.** Pozo	**Si** ❐ **1** **No**❐ **0**
**61.** Río	**Si** ❐ **1** **No**❐ **0**	**62.** Lluvia	**Si** ❐ **1** **No**❐ **0**	**63.** Estero	**Si** ❐ **1** **No**❐ **0**

64. Otras (especifique)..

65. ¿Uno de los padres /representantes/ familiares, se dedica a la agricultura o cría de animales domésticos para consumo de la casa?

Si ❐ **1** No ❐ **0**

☛ **Si la respuesta a la pregunta 65 fue SI indicar el lugar en que se lo hace:**

66. En la casa donde vive el niño(a) o sus alrededores **Si** ❐ **1** **No** ❐ **0**

67. En una finca o terreno alejado de la casa en la que vive el niño(a) **Si** ❐ **1** **No** ❐ **0**

C. Datos Generales

68. **¿Cuántos hijos son en la familia?** ....

69. **¿Cuántos hermanos mayores tiene el niño?** ……….

70. ¿Cuántos hermanos menores tiene el niño(a)? …..…...

71. ¿Qué lugar ocupa el niño(a)? ....
(llena el entrevistador, en relación a todos los hijos nacidos vivos de la madre)

72. ¿Cuántos niños (r. nacidos -15 años) **viven permanentemente en la casa?** ....

73. ¿Cuántos de los niños que viven en la casa permanentemente, son mayores que el niño(a)?
……….

Firma……………………. **Fecha…../……./200**

1. NUMERO………………….

74. ¿Cuántos de los niños que viven permanentemente en la casa, son menores que el niño(a)?
……….

75. ¿Qué lugar ocupa el niño(a) en relación a los niños que vive permanen - Te mente en la casa?
……….

76. ¿El niño(a) recibió lactancia materna? Si ❒ **1** No ❒ **0** No sabe ❒ **99**

77. Si la respuesta anterior fue <u>SI,</u> hasta qué edad lo hizo?

menos de 6 meses	❒ 1	mas de 24 meses	❒ 4
De los 6 a los 12 meses	❒ 2	No sabe	❒ 99
De los 13 a los 24 meses	❒ 3		

78. ¿Trajo el niño(a) o su representante el CARNET DE VACUNACIÓN?

Si ❒ **1** **No** ❒ **0**

☛ **Si la respuesta anterior fue <u>SI</u>, señale las dosis que se indica en el carnet:**

Vacunas que ha recibido el niño	Número de dosis de vacunas
79. BCG:	❑ 0 ❑ 1 ❑ 2
80. DTP:	❑ 0 ❑ 1 ❑ 2 ❑ 3 ❑ 4
81. SARAMPION:	❑ 0 ❑ 1
82. POLIO:	❑ 0 ❑ 1 ❑ 2 ❑ 3 ❑ 4

☛ **Si la respuesta anterior fue NO, señale las vacunas (cualquier número de dosis) que indica la madre o su representante:**

83. BCG: Si ❑ 1 No ❑ 0 No sabe ❑ 99

84. DTP: Si ❑ 1 No ❑ 0 No sabe ❑ 99

85. SARAMPION: Si ❑ 1 No ❑ 0 No sabe ❑ 99

86. POLIO: Si ❑ 1 No ❑ 0 No sabe ❑ 99

87. Tiene el niño(a) la cicatriz de la vacuna BCG? Si ❑ 1 No ❑ 0

Firma……………………. **Fecha…../……./200**

1. NUMERO…………………

88. ¿Su niño(a) fue a la guardería?: Si ❑ 1 No ❑ 0 No sabe ❑ 99

89. A qué edad fue su niño a la guardería por primera vez..........................meses

90. Si la respuesta anterior fue SI, por cuánto tiempo?........meses

☛**Vamos a hablar sobre la frecuencia con que el niño(a) come o bebe los siguientes productos:**

**Productos**	Nunca<br>**0**	A veces<br>**1**	**1** vez al mes<br>**2**	**1-4** veces por semana<br>**3**	mas de **4** veces por semana (diario)<br>**4**
**91.** CARNES (res, cerdo, pollo)					
**92.** PESCADO					
**93.** CAMARONES					

**94**. FRUTAS					
**95**. ENSALADAS					
**96**.ARROZ					
**97**. PLATANO					
**98**. YUCA					
**99**. PAN					
**100**. MANI					
**101**. PAPAS					
**102**. HUEVOS					
**103**. LECHE					
**104**. COLAS					
**105**. HAMBURGUESAS					

106. ¿El niño(a) consume leche cruda / ordeñada por lo menos 1 vez a la semana?

Si ❑ **1** No ❑ **0**

107. ¿**Durante el día cuántas horas mira la televisión el niño?**

Nunca❑ **0** A veces ❑ **1** 1-3 h ❑ **2** 4-5 h ❑ **3** más de 5 h ❑ **4**

Firma……………………. **Fecha…../……./200**

1. NUMERO…………………

108. ¿Con que frecuencia el niño(a) hace ejercicios para que le falte el aire o la respiración?

a diario ❑ **1**
3 veces por semana ❑ **2**
1 vez a la semana ❑ **3**
cada 15 días ❑ **4**
1 vez al mes ❑ **5**

☛ **¿Alguno de estos animales pasa dentro de la casa en la que vive el niño(a) ?**
(anotar si el animal pasa durante el día y/o la noche dentro de la casa en la que vive el niño)

109 Perro Si ❑ **1** No ❑ **0**

110. Gato: Si ❑ **1** No ❑ **0**

☛ **¿Alguno de estos animales pasa alrededor de la casa en la que vive el niño(a)?** (anotar si los animales están la mayor parte del tiempo en el patio o jardín, así estos animales ingresen a la casa en forma esporádica).

**111. Perro**	Si	❑ **1**	No	❑ **0**
**112. Gato**:	Si	❑ **1**	No	❑ **0**
**113. Cerdos**	Si	❑ **1**	No	❑ **0**
**114. Gallinas**	Si	❑ **1**	No	❑ **0**
**115. Otro**	Si	❑ **1**	No	❑ **0**

116. **¿El niño(a) tiene contacto con animales en fincas por lo menos una vez por semana?**

Si ❑ **1** No ❑ **0**

☛ **¿Alguna vez en la vida del niño(a), alguno de estos animales pasó /vivió dentro de la casa?**

**117. Perro**	Si	❑ **1**	No	❑ **0**
**118. Gato**	Si	❑ **1**	No	❑ **0**

Firma…………………….. **Fecha…../……./200**

1. NUMERO…………………

☛ **¿La madre del niño(a) ha presentado?**

119. Asma **Si** ❑ **1** **No** ❑ **0** **No Sabe** ❑ **99**

(algún médico le diagnosticó de ASMA o tuvo ataques de silbido al pecho 3 o más veces y por un lapso de 3 días)

120. Rinitis **Si** ❑ **1** **No** ❑ **0** **No Sabe** ❑ **99**

(algún médico le diagnosticó de rinitis alérgica, o tuvo estornudos, moqueo, picazón de la nariz o lagrimeo/ picazón de los ojos, sin estar con gripe, más de una vez)

121. Eczema Si ❑ 1 No ❑ 0 No Sabe ❑ 99

(algún médico le diagnosticó de eczema o tuvo granos o ronchas que piquen, localizados en cuello, parte anterior del codo, posterior de la rodilla, tobillos y /o nalgas que aparecen y desaparecen y que duren por lo menos 6 meses)

☛ ¿El padre del niño(a) ha presentado?

122. Asma Si ❑ 1 No ❑ 0 No Sabe ❑ 99

(algún médico le diagnosticó de ASMA o tuvo ataques de silbido al pecho 3 o más veces y por un lapso de 3 días)

123. Rinitis Si ❑ 1 No ❑ 0 No Sabe ❑ 99

(algún médico le diagnosticó de rinitis alérgica, o tuvo estornudos, moqueo, picazón de la nariz o lagrimeo/picazón de los ojos, sin estar con gripe, más de una vez)

124. Eczema Si ❑ 1 No ❑ 0 No Sabe ❑ 99

(algún médico le diagnosticó de eczema o tuvo granos o ronchas que piquen, localizados en cuello, parte anterior del codo, posterior de la rodilla, tobillos y/o nalgas que aparecen y desaparecen y que duren por lo menos 6 meses)

Firma……………………. Fecha…../……./200

1. NUMERO…………………

☛ ¿Fuma o fumaba la madre del niño(a)?

**125. En la actualidad**	Si	❑ **1**	No	❑ **0**	No sabe	❑ **99**
**126. Durante el embarazo**	Si	❑ **1**	No	❑ **0**	No sabe	❑ **99**
**127. En el primer año de vida del niño**	Si	❑ **1**	No	❑ **0**	No sabe	❑ **99**

128. ¿Actualmente fuma alguien en la casa? Si ❑ **1** No ❑ **0**

D. Migración

129. ¿Su niño(a) ha vivido en este lugar toda su vida? Si ❑ **1** No ❑ **0**

☛ **Si la respuesta anterior fue NO conteste la siguiente.**
(Para contestar esta pregunta el niño tiene que haber pasado en el lugar 3 o más meses de su vida)

	**SITIOS (localidad/ciudad/ provincia)**	**TIEMPO (meses o años)**	**Rural**	**Urbano**
**130.** Primer año de vida	**130**	**174**	**175 1❑**	**2❑**
**131.** Primer año de vida	**131**	**176**	**177 1❑**	**2❑**
**132.** De los 2 a los 5 años	**132**	**178**	**179 1❑**	**2❑**
**133.** De los 2 a los 5 años	**133**	**180**	**180 1❑**	**2❑**
**134.** Mayor que 5 años	**134**	**182**	**183 1❑**	**2❑**
**135.** Mayor que 5 años	**135**	**184**	**185 1❑**	**2❑**

136 ¿El niño(a) tiene familiares viviendo en Guayaquil? Si ❑**1** No ❑**0**

137. Si la respuesta anterior fue SI, sabe la dirección (localidad/ barrio)?

**Relación Familiar**	**Dirección (localidad/ barrio)**

Firma……………………. **Fecha…../……./200**

1. NUMERO…………………

138. ¿El niño(a) tiene familiares viviendo en la ciudad de Esmeraldas?

Si ❑ **1** No ❑ **0**

139. Si la respuesta es SI, sabe la dirección (localidad/ barrio)?

**Relación Familiar**	**Dirección (localidad/ barrio)**

E. Tratamiento antiparasitario

140. **¿Cuándo fue la última vez que el niño(a) tomó purgante?** ....meses

141. ¿Sabe el nombre del medicamento?___________________

F. Silbido

142. **¿Alguna vez en la vida, el niño(a) tuvo silbido al pecho, (en cualquier época del pasado)?**

Si ❑ 1 No ❑ 0 No Sabe ❑ 99

143. **¿El niño(a) ha tenido silbido al pecho en los últimos 12 meses (último año)?**

Si ❑ 1 No ❑ 0 No Sabe ❑ 99

144. ¿Cuántos ataques o crisis de silbido al pecho, ha tenido el niño(a) en estos últimos 12 meses (último año)?

Ninguno ❑ **0** 1 a 3 ❑ **1** 4 a 12 ❑ **2** más de 12 ❑ **3**

145. ¿En estos últimos 12 meses (último año) cuántas veces el niño(a) se ha despertado en la noche debido al silbido al pecho?

Nunca ha despertado con silbido ❑ **0**

Menos de una noche por semana ❑ **1**

Una o más noches por semana ❑ **2**

Firma……………………. Fecha…../……./200

1. NUMERO…………………

146. ¿En estos últimos 12 meses (último año) ha sido el silbido al pecho tan severo (o tan fuerte) como para no dejarlo hablar más de una o dos palabras entre cada respiración?

Si ❑ 1 No ❑ 0 No Sabe ❑ 99

147. ¿En estos últimos 12 meses (último año), el niño(a) ha tenido silbido al pecho durante o después de hacer ejercicio (correr, trabajar, etc.)?

Si ❑ 1 No ❑ 0 No Sabe ❑ 99

148. ¿Alguna vez en la vida el niño(a) fue diagnosticado de asma o bronquitis por un médico?

Si ❑ 1 No ❑ 0 No Sabe ❑ 99

149. ¿En estos últimos 12 meses (último año), el niño(a), ha tenido tos seca en la noche? (aparte de la asociada con resfríos, gripes, o a infecciones respiratorias)

Si ❑ 1 No ❑ 0 No Sabe ❑ 99

G. <u>Rinitis</u>

150. ¿Alguna vez en su vida, el niño(a), ha tenido problemas de estornudo, goteo de la nariz (moqueo), o que se le tape la nariz? (cuando no tenía resfrío común ni gripe)

Si ❑ 1 No ❑ 0 No Sabe ❑ 99

151. ¿En los últimos 12 meses, el niño, tuvo problemas de estornudo, goteo de la nariz (moqueo), o que se le tape la nariz? (cuando no tenía resfrío común ni gripe)

Si ❑ 1 No ❑ 0 No Sabe ❑ 99

152. ¿En los últimos 12 meses, al niño(a), junto con el problema de la nariz ¿le picaban y lloraban los ojos?

Si ❑ 1 No ❑ 0 No Sabe ❑ 99

Firma……………………. Fecha…../……./200

1. NUMERO…………………

153. ¿En los últimos 12 meses (último año) en que cantidad interfirieron estos problemas nasales con las actividades diarias del niño(a)?

En nada ❑ **0** Un poco ❑ **1** Moderadamente ❑ **2** Mucho ❑ **3**

154. ¿Alguna vez en la vida el niño(a) fue diagnosticado por un médico de rinitis alérgica?

Si ❑ 1 No ❑ 0 No Sabe ❑ 99

H. Eczema

155. ¿El niño(a) ha tenido alguna vez en la vida granos o ronchas acompañados de picazón, que aparezcan y desaparezcan durante por lo menos 6 meses?

Si ❑ 1 No ❑ 0 No Sabe ❑ 99

156. ¿El niño(a) ha atenido éstas ronchas o granos con picazón en algún momento de estos últimos 12 meses ?

Si ❑ 1 No ❑ 0 No Sabe ❑ 99

☛¿Los granos o ronchas con picazón le han salido en alguno de los siguientes lugares?

	SI	NO
**157. Alrededor de los ojos**..	❑ 1	❑ 0
**158. Alrededor o parte frontal del cuello**................	❑ 1	❑ 0
**159. En las orejas**..	❑ 1	❑ 0

160. Parte frontal de los codos................................. ❑1 ❑0

161. Detrás de las rodillas.. ❑1 ❑0

162. Parte frontal de los tobillos............................... ❑1 ❑0

163. Nalgas... ❑1 ❑0

164. ¿A qué edad aparecieron los granos o ronchas por primera vez en el niño(a)?

____

Firma……………………. Fecha…../……./200

1. NUMERO…………………

165. ¿Ha estado completamente sano de este problema de la piel (granos o ronchas con picazón) en algún momento en los últimos 12 meses (último año)?

Si ❑1 No ❑0

166. ¿Cuántas veces en los últimos 12 meses (último año) (en promedio) se ha mantenido despierto el niño durante la noche debido a la picazón con granos o ronchas?

Ninguna vez en los últimos 12 meses **❑0**

Menos de una noche por semana **❑1**

Una o más noches por semana **❑2**

167. ¿Alguna vez un médico le ha diagnosticado al niño(a) de eczema o dermatitis atópica?

Si ❑ 1 No ❑0 No Sabe ❑99

I. <u>Fuente</u> <u>de</u> <u>información</u>:

168. Niño **Si❑1 No❑0** **169.** Tía (o) **Si❑1 No❑0**

**170**. Hermana (o)	**Si❑ 1 No❑ 0**	**171.** Padre	**Si❑ 1 No❑ 0**
**172**. Madre	**Si❑ 1 No❑ 0**	**173.** Representante	**Si❑ 1 No❑ 0**
