# Supplementary material for: Hygiene, atopy and wheeze–eczema–rhinitis symptoms in schoolchildren from urban and rural Ecuador
Source: Thorax. 2013 Oct 8;69(3):232–9. doi: 10.1136/thoraxjnl-2013-203818 (PMC3932750; doi:10.1136/thoraxjnl-2013-203818)
Supplement: Web tables [file thoraxjnl-2013-203818-s2.pdf]

		SPT				Wheeze				Rhinitis				Eczema			
		No		Yes		No		Yes		No		Yes		No		Yes	
		N	%	N	%	N	%	N	%	N	%	N	%	N	%	N	%
Pets inside home	No	2240	38.6%	311	40.9%	2355	39.3%	242	37.2%	2460	39.4%	155	32.8%	2513	39.3%	121	33.6%
	Yes	3565	61.4%	449	59.1%	3641	60.7%	409	62.8%	3791	60.6%	318	67.2%	3878	60.7%	239	66.4%
Crowding	< 3	1768	30.5%	271	35.6%	1845	30.8%	204	31.4%	1928	30.9%	161	34.2%	1972	30.9%	121	33.6%
	>=3	4031	69.5%	490	64.4%	4149	69.2%	445	68.6%	4320	69.1%	310	65.8%	4414	69.1%	239	66.4%
Birth order	<5th	4234	72.9%	531	70.0%	4311	71.9%	503	77.4%	4496	71.9%	380	80.5%	4614	72.2%	275	76.6%
	>=5th	1571	27.1%	228	30.0%	1686	28.1%	147	22.6%	1756	28.1%	92	19.5%	1778	27.8%	84	23.4%
Bathroom	Field	1482	25.5%	181	23.8%	1497	25.0%	185	28.4%	1557	24.9%	144	30.4%	1636	25.6%	78	21.7%
	Others	4324	74.5%	580	76.2%	4501	75.0%	466	71.6%	4696	75.1%	329	69.6%	4757	74.4%	282	78.3%
Potable drinking water	No	3580	61.6%	504	66.2%	3711	61.9%	432	66.3%	3907	62.5%	279	59.0%	3993	62.4%	222	61.7%
	Yes	2229	38.4%	257	33.8%	2289	38.2%	220	33.7%	2349	37.5%	194	41.0%	2403	37.6%	138	38.3%
Attended daycare	No	3155	55.0%	419	55.9%	3270	55.1%	356	55.4%	3419	55.4%	249	52.9%	3505	55.5%	176	49.3%
	Yes	2577	45.0%	330	44.1%	2667	44.9%	287	44.6%	2758	44.6%	222	47.1%	2812	44.5%	181	50.7%
House construction	others	2825	48.6%	345	45.3%	2863	47.7%	320	49.1%	3018	48.2%	215	45.5%	3070	48.0%	171	47.5%
	bamboo/wood	2984	51.4%	416	54.7%	3137	52.3%	332	50.9%	3238	51.8%	258	54.5%	3326	52.0%	189	52.5%
Father engaged in agriculture	No	3907	68.7%	434	58.6%	3947	67.1%	445	69.7%	4120	67.3%	334	71.5%	4252	67.9%	221	63.0%
	Yes	1783	31.3%	307	41.4%	1935	32.9%	193	30.3%	2000	32.7%	133	28.5%	2011	32.1%	130	37.0%
Contact with farm animals*	No	4545	78.3%	548	72.1%	4650	77.6%	502	77.1%	4871	78.0%	347	73.4%	4982	78.0%	259	71.9%
	Yes	1257	21.7%	212	27.9%	1343	22.4%	149	22.9%	1377	22.0%	126	26.6%	1406	22.0%	101	28.1%
Unpasteurized milk†	No	3478	60.0%	431	56.9%	3580	59.8%	382	58.6%	3717	59.5%	279	59.0%	3821	59.9%	190	52.8%
	Yes	2321	40.0%	326	43.1%	2409	40.2%	270	41.4%	2525	40.5%	194	41.0%	2561	40.1%	170	47.2%
Any geohelminth	No	2221	39.4%	363	49.7%	2361	41.0%	238	38.1%	2432	40.5%	192	41.9%	2493	40.6%	144	41.3%
	Yes	3410	60.6%	367	50.3%	3404	59.0%	387	61.9%	3573	59.5%	266	58.1%	3646	59.4%	205	58.7%
A. lumbricoides	No	3670	65.2%	530	72.6%	3802	65.9%	411	65.8%	3940	65.6%	319	69.7%	4049	66.0%	228	65.3%
	Yes	1961	34.8%	200	27.4%	1963	34.1%	214	34.2%	2065	34.4%	139	30.3%	2090	34.0%	121	34.7%

T. trichiura	No	2897	51.4%	459	62.9%	3078	53.4%	311	49.8%	3178	52.9%	235	51.3%	3228	52.6%	205	58.7%
	Yes	2734	48.6%	271	37.1%	2687	46.6%	314	50.2%	2827	47.1%	223	48.7%	2911	47.4%	144	41.3%
Hookworm	No	5351	95.0%	681	93.3%	5471	94.9%	590	94.4%	5690	94.8%	440	96.1%	5827	94.9%	325	93.1%
	Yes	280	5.0%	49	6.7%	294	5.1%	35	5.6%	315	5.2%	18	3.9%	312	5.1%	24	6.9%

Supplementary Table 1. Frequencies of poor hygiene and farming exposures according to the presence and absence of allergen skin test reactivity (SPT), and recent symptoms of wheeze, rhinitis, and eczema in urban and rural schoolchildren combined. *Pigs, cows, horses, mules, donkeys. **†** - Consumption of unpasteurized milk at least once weekly. Median crowding was 3 persons per sleeping room.

	SPT<br>OR (95% CI)<br>p value		Inter.<br>P<br>value	Wheeze<br>OR (95% CI)<br>p value		Inter.<br>P<br>value	Rhinitis<br>OR (95% CI)<br>p value		Inter.<br>P<br>value	Eczema<br>OR (95% CI)<br>p value		Inter.<br>P<br>value
Hygiene exposure	Urban	Rural		Urban	Rural		Urban	Rural		Urban	Rural	
Pets inside home<br>Yes vs. No	0.75(0.57-0.98)<br>0.035	1.08(0.88-1.32)<br>0.451	0.036	1.16(0.88-1.55)<br>0.291	1.07(0.86-1.32)<br>0.560	0.616	1.53(1.12-2.10)<br>0.007	1.22(0.93-1.60)<br>0.143	0.245	1.05(0.75-1.47)<br>0.779	1.37(1-1.87)<br>0.053	0.229
Crowding<br>≥3 vs. <3	0.73(0.55-0.96)<br>0.025	0.89(0.72-1.09)<br>0.261	0.297	1.03(0.77-1.39)<br>0.829	0.88(0.70-1.10)<br>0.254	0.421	1.02(0.74-1.40)<br>0.901	0.77(0.59-1.01)<br>0.063	0.290	1.01(0.71-1.44)<br>0.942	0.84(0.62-1.15)<br>0.285	0.620
Birth order<br>≥5th vs. ≤4th	0.83(0.57-1.20)<br>0.317	1.16(0.95-1.41)<br>0.145	0.105	**1.13(0.79-1.61)**<br>**0.497**	**0.63(0.50-0.80)**<br>**<0.001**	**0.007**	0.80(0.53-1.22)<br>0.304	0.59(0.44-0.79)<br><0.001	0.292	0.97(0.62-1.52)<br>0.894	0.75(0.55-1.03)<br>0.079	0.413
Bathroom<br>Field vs. others	0.67(0.37-1.24)<br>0.202	0.92(0.74-1.14)<br>0.443	0.368	0.77(0.43-1.38)<br>0.382	1.17(0.93-1.47)<br>0.179	0.187	0.86(0.47-1.58)<br>0.630	1.78(1.35-2.35)<br><0.001	0.032	0.53(0.23-1.21)<br>0.131	1.06(0.76-1.48)<br>0.722	0.095
Potable drinking water<br>No vs. Yes	0.87(0.48-1.57)<br>0.648	0.80(0.45-1.45)<br>0.477	0.896	1.25(0.71-2.20)<br>0.446	2.10(1.13-3.90)<br>0.019	0.167	1.29(0.75-2.22)<br>0.363	2.66(1.24-5.72)<br>0.012	0.288	1.25(0.73-2.14)<br>0.420	2.78(0.92-8.39)<br>0.069	0.146
Attended daycare<br>Yes vs. No	1.17(0.89-1.53)<br>0.263	0.83(0.67-1.02)<br>0.073	0.046	0.91(0.69-1.21)<br>0.530	0.97(0.78-1.19)<br>0.767	0.689	1.50(1.12-2.0)<br>0.007	0.95(0.73-1.24)<br>0.716	0.030	1.40(1-1.96)<br>0.047	1.19(0.88-1.61)<br>0.247	0.522
House construction<br>Wood/bamboo vs. others	1(0.74-1.37)<br>0.954	1.07(0.86-1.32)<br>0.565	0.846	0.93(0.68-1.28)<br>0.669	0.86(0.68-1.07)<br>0.179	0.690	1.14(0.83-1.59)<br>0.412	1.52(1.13-2.05)<br>0.006	0.254	0.87(0.59-1.27)<br>0.468	1.28(0.91-1.80)<br>0.150	0.080
Father engaged in agriculture<br>Yes vs. No	0.94(0.53-1.68)<br>0.844	1.25(0.99-1.57)<br>0.057	0.367	0.96(0.53-1.74)<br>0.899	0.85(0.68-1.05)<br>0.144	0.672	1.21(0.68-2.15)<br>0.514	1(0.75-1.34)<br>0.980	0.479	1.20(0.61-2.33)<br>0.600	1.11(0.79-1.55)<br>0.546	0.972
Contact with farm animals*<br>Yes vs. No	0.89(0.54-1.46)<br>0.644	1.25(1-1.55)<br>0.042	0.203	1.02(0.63-1.66)<br>0.931	1.06(0.85-1.34)<br>0.599	0.908	1.86(1.21-2.85)<br>0.005	1.42(1.07-1.87)<br>0.015	0.194	2.14(1.34-3.41)<br>0.002	1.29(0.94-1.78)<br>0.118	0.063
Unpasteurized milk†<br>Yes vs. No	1.16(0.88-1.53)<br>0.283	0.96(0.77-1.19)<br>0.683	0.257	0.88(0.66-1.18)<br>0.394	1.18(0.96-1.48)<br>0.118	0.107	0.85(0.63-1.16)<br>0.312	1.23(0.93-1.61)<br>0.143	0.081	0.89(0.62-1.25)<br>0.494	1.46(1.05-2)<br>0.021	0.029
Any geohelminth<br>Yes vs. No	0.78(0.59-1.05)<br>0.109	0.58(0.47-0.72)<br><0.001	0.099	1.24(0.93-1.64)<br>0.136	0.97(0.76-1.22)<br>0.770	0.216	0.86(0.63-1.16)<br>0.327	1.19(0.88-1.61)<br>0.246	0.135	0.95(0.67-1.34)<br>0.765	1.30(0.92-1.84)<br>0.132	0.118
A. lumbricoides<br>Yes vs. No	0.60(0.40-0.91)<br>0.015	0.69(0.56-0.87)<br>0.001	0.556	0.74(0.51-1.09)<br>0.130	1.06(0.86-1.32)<br>0.571	0.101	0.62(0.40-0.94)<br>0.027	1.09(0.84-1.44)<br>0.502	0.033	0.93(0.60-1.43)<br>0.738	1.20(0.88-1.65)<br>0.252	0.206
T. trichiura<br>Yes vs. No	0.81(0.60-1.10)<br>0.181	0.59(0.48-0.73)<br><0.001	0.091	1.40(1.05-1.87)<br>0.020	0.95(0.76-1.19)<br>0.651	0.044	0.98(0.72-1.34)<br>0.892	1.17(0.88-1.54)<br>0.282	0.351	1.01(0.71-1.44)<br>0.938	0.89(0.65-1.23)<br>0.504	0.868
Hookworm<br>Yes vs. No	1.18(0.57-2.45)<br>0.658	1.14(0.76-1.70)<br>0.526	0.975	1.69(0.84-3.41)<br>0.138	0.90(0.54-1.48)<br>0.683	0.216	0.91(0.43-1.90)<br>0.803	0.80(0.41-1.58)<br>0.520	0.813	0.50(0.16-1.58)<br>0.238	1.76(1.02-3.03)<br>0.042	0.011

Supplementary Table 2. Associations between study outcomes and poor hygiene/farming exposures stratified by área of residence. Odds ratios (ORs) and 95% confidence intervals (95% CI) were calculated using random effects logistic regression in which clustering by rural community or urban neighbourhood was adjusted. Age and sex were included as *a priori* confounders and ethnicity, maternal educational level, household income and body mass index were considered as potential confounders and adjusted for where they had a significant effect on study outcomes. Interactions (inter.) with P≤0.01 were considered relevant.

*Pigs, cows, horses, mules, donkeys. **†** - Consumption of unpasteurized milk at least once weekly. Median crowding was 3 persons per sleeping room.

Hygiene exposure	Wheeze<br>OR (95% CI)<br>P value		Inter. P value	Rhinitis<br>OR (95% CI)<br>P value		Inter. P value	Eczema<br>OR (95% CI)<br>P value		Inter. P value
	SPT-	SPT+		SPT-	SPT+		SPT-	SPT+	
Pets inside home<br>Yes vs. No	1.08 (0.89-1.30)<br>0.449	1.30 (0.84-2.01)<br>0.245	0.470	1.54 (1.23-1.93)<br>0.001	0.80 (0.46-1.37)<br>0.410	0.019	1.27 (0.98-1.64)<br>0.071	1.22 (0.67-2.24)<br>0.516	0.724
Crowding<br>≥3 vs. <3	0.94 (0.77-1.15)<br>0.573	1.07 (0.69-1.67)<br>0.760	0.618	0.89 (0.71-1.11)<br>0.301	0.98 (0.55-1.74)<br>0.941	0.786	0.86 (0.67-1.12)<br>0.259	0.85 (0.46-1.59)<br>0.629	0.989
Birth order<br>≥5th vs. ≤4th	0.75 (0.60-0.93)<br>0.010	0.75 (0.47-1.23)<br>0.261	0.987	0.65 (0.50-0.84)<br>0.001	0.64 (0.32-1.25)<br>0.191	0.675	0.81 (0.61-1.08)<br>0.157	0.64 (0.32-1.27)<br>0.201	0.716
Bathroom<br>Field vs. others	1.18 (0.94-1.47)<br>0.147	0.97 (0.59-1.69)<br>0.899	0.355	1.53 (1.19-1.95)<br>0.001	0.80 (0.38-1.68)<br>0.550	0.027	0.92 (0.68-1.26)<br>0.616	0.90 (0.44-1.86)<br>0.778	0.768
Potable drinking water<br>No vs. Yes	**1.42 (1.13-1.79)**<br>**0.002**	**0.74 (0.47-1.14)**<br>**0.172**	**0.010**	**1.05 (0.79-1.40)**<br>**0.741**	**0.48 (0.26-0.88)**<br>**0.019**	**0.010**	0.99 (0.70-1.39)<br>0.949	0.87 (0.39-1.94)<br>0.726	0.623
Attended daycare<br>Yes vs. No	0.99 (0.82-1.19)<br>0.888	0.87 (0.56-1.33)<br>0.510	0.588	1.15 (0.93-1.42)<br>0.202	1.48 (0.85-2.57)<br>0.163	0.471	1.33 (1.04-1.70)<br>0.025	1.12 (0.62-2.02)<br>0.712	0.455
House construction<br>Wood/bamboo vs. others	1.01 (0.84-1.23)<br>0.904	0.59 (0.38-0.90)<br>0.014	0.018	**1.37 (1.09-1.72)**<br>**0.008**	**0.66 (0.36-1.21)**<br>**0.179**	**0.005**	1.03 (0.77-1.38)<br>0.858	0.79 (0.41-1.50)<br>0.466	0.462
Father engaged in agriculture<br>Yes vs. No	0.95 (0.77-1.18)<br>0.663	0.77 (0.49-1.20)<br>0.247	0.465	0.87 (0.67-1.13)<br>0.285	1.04 (0.53-2.04)<br>0.920	0.775	1.03 (0.77-1.38)<br>0.858	1.55 (0.77-3.12)<br>0.220	0.484
Contact with farm animals*<br>Yes vs. No	1.07 (0.85-1.34)<br>0.561	0.93 (0.57-1.51)<br>0.770	0.658	1.36 (1.05-1.75)<br>0.018	1.73 (0.92-3.26)<br>0.087	0.789	1.48 (1.11-1.97)<br>0.008	1.18 (0.61-2.32)<br>0.615	0.703
Unpasteurized milk†<br>Yes vs. No	1.05 (0.87-1.28)<br>0.584	1.08 (0.71-1.65)<br>0.718	0.892	1.02 (0.82-1.27)<br>0.856	1.18 (0.68-2.07)<br>0.560	0.666	1.12 (0.86-1.44)<br>0.396	1.47 (0.80-2.70)<br>0.218	0.663
Any geohelminth<br>Yes vs. No	1.22 (1.00-1.49)<br>0.055	0.87 (0.57-1.35)<br>0.543	0.130	1.02 (0.81-1.28)<br>0.877	0.95 (0.54-1.67)<br>0.860	0.675	1.01 (0.78-1.31)<br>0.919	1.32 (0.71-2.47)<br>0.384	0.376
A. lumbricoides<br>Yes vs. No	1.00 (0.82-1.23)<br>0.971	1.25 (0.77-2.02)<br>0.362	0.487	0.83 (0.65-1.05)<br>0.116	1.50 (0.79-2.82)<br>0.212	0.202	0.94 (0.72-1.24)<br>0.679	1.88 (0.98-3.63)<br>0.058	0.062
T. trichiura<br>Yes vs. No	1.23 (1.01-1.49)<br>0.037	0.85 (0.54-1.34)<br>0.472	0.094	1.14 (0.92-1.43)<br>0.235	0.78 (0.43-1.43)<br>0.431	0.194	0.86 (0.66-1.12)<br>0.257	0.96 (0.51-1.82)<br>0.907	0.690
Hookworm<br>Yes vs. No	1.09 (0.70-1.69)<br>0.700	1.21 (0.52-2.80)<br>0.658	0.854	0.64 (0.34-1.19)<br>0.156	2.00 (0.74-5.46)<br>0.174	0.057	1.21 (0.70-2.09)<br>0.490	0.96 (0.33-3.04)<br>0.991	0.884

Supplementary Table 3. Associations between study outcomes and poor hygiene/farming exposures stratified by allergen skin prick test reactivity (SPT). Odds ratios (ORs) and 95% confidence intervals (95% CI) were calculated using random effects logistic regression in which clustering by rural community or urban neighbourhood was adjusted. Age and sex were included as *a priori* confounders and ethnicity, maternal educational level, household income and body mass index were considered as potential confounders and adjusted for where they had a significant effect on study outcomes. Interactions (inter.) with P≤0.01 were considered relevant.

*Pigs, cows, horses, mules, donkeys. **†** - Consumption of unpasteurized milk at least once weekly. Median crowding was 3 persons per sleeping room.
